# Self-management challenges and support needs among patients with primary glaucoma: a qualitative study

**DOI:** 10.1186/s12912-023-01527-y

**Published:** 2023-11-14

**Authors:** Yiting Hua, Hujie LU, Jingyao Dai, Yewei Zhou, Wenzhe Zhou, Aisun Wang, Yanyan Chen, Youping Liang

**Affiliations:** 1 School of Ophthalmology and Optometry, Wenzhou Medical University, 270 West Xueyuan Road, Wenzhou, Zhejiang 325027 China; 2 National Clinical Research Center for Ocular Diseases, Eye Hospital, Wenzhou Medical University, 270 West Xueyuan Road, Wenzhou, Zhejiang 325027 China

**Keywords:** Glaucoma, Self-management, Self‑management support, Needs, Qualitative research

## Abstract

**Background:**

Self-management plays an important role in the disease management of glaucoma patients. The effectiveness of the program can be improved by assessing the patient’s perspective and needs to tailor self-management support. Most studies have focused on assessing one of these self-management behaviours, such as medication adherence, and there is a lack of systematic assessment of the support needs and challenges of self-management for patients with glaucoma. Therefore, in this study, we conducted an in-depth investigation into the self-management challenges and support needs of patients with primary glaucoma, providing a basis for nursing staff to implement self-management support.

**Method:**

The phenomenological method and semistructured interviews were used in this study. A total of 20 patients with primary glaucoma were recruited between June and December 2022. Colaizzi’s analysis method was used to analyse the interview data.

**Results:**

Challenges for patients include becoming an expert in glaucoma, managing negative emotions, adapting to daily life changes and resuming social activities. To address these challenges, four themes of patient self-management support needs were identified: (1) health information support, (2) social support, (3) psychological support, and (4) daily living support.

**Conclusion:**

Patients with primary glaucoma experience varying degrees of challenge in dealing with medical, emotional, and social aspects. Comprehending the support needs of patients, healthcare professionals should deliver targeted, personalized and comprehensive self-management interventions to enhance their capacity of patients to perform self-management and improve their quality of life.

**Supplementary Information:**

The online version contains supplementary material available at 10.1186/s12912-023-01527-y.

## Background

As the leading cause of global irreversible blindness, glaucoma poses an important global health challenge [1, 2]. Glaucoma is managed as a chronic disease by consensus among ophthalmologists due to its incurable nature [3]. The reality that patients are being asked to become more actively involved in their chronic conditions means that responsibility for day-to-day disease management is gradually shifting from the healthcare professional to the individual [4]. Self-management refers to an individual’s ability to manage the symptoms, treatment, physical and psychosocial consequences and lifestyle changes inherent in a chronic condition [5]. Good self-management includes the ability to monitor one’s condition and influence the cognitive, behavioural and emotional responses necessary to maintain a satisfactory quality of life. In this way, a dynamic and continuous process of self-regulation is established, they will encounter various challenges in managing themselves. For patients with glaucoma, medical management of the disease, physical function promotion and the vision-related life adjustments may be challenging when it comes to self-management [6, 7].

Increasing evidence suggests that effective self-management is crucial for optimizing patient health outcomes and enhancing their quality of life [8–10]. Improving patients’ self-management ability is a pressing issue that needs to be addressed [11, 12]. Self-management support (SMS) [13] refers to the systematic provision of health education and supportive measures by healthcare professionals to empower patients in managing their own health. This includes regular assessment of disease progression and problems, setting management goals, and problem-solving support measures to enhance skills and confidence in managing health problems. SMS has been utilized in the management of chronic diseases such as COPD and diabetes [14–16], demonstrating effectiveness in enhancing biomedical indicators and quality of life, facilitating long-term illness management, and devising solutions to health-related issues.

Tailoring SMS to the individualized needs of patients may enhance the efficacy of the program [17]. Previous studies have shown the importance of assessing patients’ perspectives or needs for the implementation of self-management in patients with chronic diseases [11, 18–20]. Several studies [21–23] have also reported on the assessments of glaucoma patients with self-management disorders, mostly focusing on medication adherence or a particular aspect. Before we apply specific self-management support strategies, it is necessary to conduct a comprehensive evaluation of the glaucoma patient. However, some other relevant self-management behaviours have not been examined widely and an assessment of the support needs and challenges of self-management for people with glaucoma is lacking. Therefore, to enhance glaucoma patients’ ability to manage their condition effectively, this study employs phenomenological research methods and qualitative interviews to comprehensively investigate the challenges of self-management and support needs.

## Materials and methods

### Design

We employed descriptive phenomenological approach, which is rooted in Husserl’s philosophy and emphasizes the process of “returning to the thing itself”. Its objective is to investigate individuals’ lived experiences, thereby uncovering their perspectives and needs [24]. This study focuses on the self-management experience and support needs of people with glaucoma and therefore adopted descriptive phenomenological approach.

### Ethics statement

Ethical approval for this study was granted by the ethics committee of the Eye Hospital of Wenzhou Medical University (Study-ID: 2021-056-K-48). Prior to the commencement of data collection, all participants in the study were provided with written informed consent and gave written permission to be interviewed and audiotaped. They had the right to withdraw from the study at any point during the interview period.

### Participants and sampling

Glaucoma patients were voluntarily recruited from the Eye Hospital of Wenzhou Medical University. This study utilizes a purposeful sampling method in conjunction with a maximum variation sampling approach. The sample size of this study is based on data saturation.

The inclusion criteria were: (1) patients diagnosed with primary glaucoma, (2) had been treated with drugs, surgery, or lasers, (3) age ≥ 18 years, and (4) had certain understanding and expression ability. Exclusion criteria were (1) no other acute or chronic eye diseases, (2) no concurrent serious physical diseases and (3) no other psychiatric diseases.

### Data collection

Participants were recruited from June to December 2022. This study was conducted in the inpatient department, with interviews taking place in a serene and uninterrupted ward or conference room. The interview guide was revised based on the literature review and the results of the preinterview of 2 interviewees (Table 1). We made contact with the participants prior to the formal interviews, introducing ourselves and explaining the aim of the research project. During the interview, patients were audio recorded throughout the entire process, while nonverbal data such as facial expressions and body movements were documented in the interview notes. The duration of each interview was regulated to fall within the range of 20 to 40 min and adjusted based on the specific circumstances of the interviewees.

**Table 1:** Semistructured interview guide

Semistructured interview guide
1. What is the reason for your admission to the hospital?
2. How has your life been impacted since receiving a diagnosis of glaucoma?
3. What strategies or techniques do you employ to effectively manage your illness?
4. What difficulties do you currently have in managing your disease?^a^
5. What help have you received? What type of assistance do you require and in what manner?

^a^Including medication, diet, sleep, exercise and so on

### Qualitative analysis

The recording was transcribed and categorized by two researchers within 24 h. Colizzi’s seven-step method [25] was used for data analysis in this study: (1) reading and rereading the interview transcripts of all glaucoma participants; (2) extracting meaningful statements related to self-management challenges and support needs; (3) formulating more general restatements or meanings for each significant statements extracted from the text; (4) classifying the codes into themes and subthemes; (5) merging the formed themes with the research content and describing them in detail; (6) removing redundant descriptions and stating the basic structure of self-management challenges and support needs for people with glaucoma; and (7) returning to the research object for verification. Based on reflection, two researchers (Hua Y.T. and Lu H.J.) summarized the data analysis results, and the analysis results of the other researcher (Dai J.Y.) were compared with those of the two researchers. The information was reconstructed in a specific sequence and focus through joint discussion, resulting in a cohesive theme with internal correlation. The data were analysed using NVivo 12.0.

### Rigour

Credibility, dependability, conformability and transferability are terms that have been used to define different dimensions of trustworthiness in qualitative research. To add credibility, participants with different perspectives, ages and genders were selected to help us better understand the phenomenon we were studying. Both interviewers were female ophthalmic nurses with some training in qualitative research methods and clinical experience. During the interview, the authors strive to retain their pre-understanding to find something new. Researchers adopt a neutral stance, refrain from interrupting participants arbitrarily, provide timely non-judgmental responses, adapt questions based on the context, pursue and clarify ambiguous inquiries, and avoid using leading prompts. In addition, to identify the themes and subthemes of this study, we sought the agreement of co-researchers, experts and participants. In qualitative research, there can be no dependability without credibility, the recordings were transcribed verbatim, and the detailed transcriptions enhanced the dependability of the study. The collection, analysis and interpretation of the data were continuously reviewed and detailed to ensure their reliability. Regarding transferability, this study explained the inclusion criteria, exclusion criteria and general demographic characteristics. The COREQ guidelines were followed in reporting the methods (Supplement 1).

## Results

A total of 21 participants were enrolled, with one participant withdrawing from the interview due to emotional distress during the process, resulting in a final sample size of 20 patients. After interviewing 15 participants, we considered that the study data had reached thematic saturation, but continued to conduct five additional interviews, analysing no additional themes to confirm data saturation. The 20 voluntary participants were between 18 and 70 years of age. The general characteristics of the participants are presented in Table 2.

**Table 2:** Characteristics of Participants (N = 20)

Characteristic	N(%)
Gender	
Male	10 (50%)
Female	10 (50%)
Age	
≤ 55	9 (45%)
>55	11 (55%)
Education	
Junior high school and less	15 (75%)
High school or higher	5 (25%)
Marital status	
Married	14 (70%)
Others^a^	6 (30%)
Glaucoma type^b^	
PACG	10 (50%)
POAG	10 (50%)
Duration of diagnosis	
<1 year	4(20%)
1–5 years	9(45%)
>5 years	7(35%)

Note: ^a^Including unmarried, divorced, widows, and widowers

^b^PACG = primary angle-closure glaucoma; POAG = primary open-angle glaucoma

### Self-management challenges postillness

Patients reported a range of challenges following their diagnosis with glaucoma, encompassing medical, psychological, and social difficulties. They illustrated distinct illness-related issues from diverse experiences. For instance, in the early stages of glaucoma, most patients lacked knowledge regarding the disease’s characteristics and proper eye drop application techniques. *" I have not even heard of it, not even once” (P2); “I did not get it before, but now I know you should wait a bit after using eye drops” (P7)*; There were difficulties in regulating emotions; patients experienced anxiety, irritability, and reduced social interaction while struggling to accept the impact of hypopsia and visual field defects. *“It is quite bothersome that my visual acuity is poor” (P9, 16); “I used to derive pleasure from travelling, but due to the necessity of refrigerating eye drops, my inclination towards travelling has gradually diminished” (P3);* There were difficulties coping with medical regimens in daily life; some patients were unsure how to handle adverse symptoms and others struggled with monitoring intraocular pressure (IOP) changes. *“I do not know which professional to consult if there are any issues such as infections after my surgery” (P10, 18); “The monitoring of IOP requires hospital visits and cannot be measured during lunch breaks” (P13);* In summary, numerous self-management challenges were identified: becoming an expert in glaucoma, managing negative emotions, adapting to daily life changes and resuming social activities.

### Support needs

The support needs of patients vary based on age, education, illness progression, and medical conditions. When patients encounter more challenges, their need for support is greater, and the longer the disease progresses, the less they need. The thematic analysis revealed four main themes and eleven subthemes reflecting support needs of patients (Table 3). The four support needs related to self-management have been summarized and reported.

**Table 3:** Theme and subthemes

theme	subthemes
Health information support	Providing personal knowledge and guidance
	Prompting shared decision making
	Using mobile medical technologies
	Strengthen humanistic care
Social support	Family support
	Peer support
	Financial support
Psychological support	Managing negative emotions
	Need to maintain self-esteem
Daily living support	Guiding symptom monitoring
	Provides portable ways to monitor IOP

### Theme 1: health information support

#### Providing personal knowledge and guidance

Becoming an expert in the field of glaucoma was considered a challenge. To achieve this objective, patients sought to improve their management skills with guidance from healthcare professionals. “Infusion” or “one-size-fits-all” education was not preferred by patients, who generally favoured more targeted education.

*“I already got the gist of the lecture in the ward. What I’m truly curious about are the precautions for surgery, cause everyone’s unique, right?” (P7)*.

After being diagnosed with glaucoma, many patients have actively sought medical information related to the disease to understand its characteristics and prognosis. However, a greater number of patients expressed a desire to obtain authoritative information from healthcare professionals. Furthermore, the complex medical aspects of glaucoma can be challenging for patients to comprehend, leading to their complaints. Instead of utilizing obscure medical terminology, it is advisable to condense the most important information and convert it into easily comprehensible and memorable phrases.

*“We can find tons of information (online), but we’d rather get it from the hospital.” (P1)*.

*“I’m too old to know the difference between open-angle glaucoma and closed-angle glaucoma… Just tell me how to use these (eye drops), okay? It should be as easy to remember as a schoolkid’s times table.” (P8)*.

#### Prompting shared decision making

With the shift towards patient-centred medicine, relying solely on clinicians to make treatment decisions for glaucoma patients is inadequate in meeting the demands of modern medical diagnosis. Therefore, it is concerning that patients express a strong desire to participate in surgical decision-making and medical treatment. Due to the issues of information asymmetry and limited communication time between medical professionals and patients, the hospital’s support of patient decision aids, which provide the pros and cons of various treatment options to enhance decision-making efficiency, was deemed necessary.

*“You should hook us up with a brochure that breaks down the success rate and prognosis of the operation, cause that is what our patients care about the most. Mostly, doctors make their own calls on which surgical options to take. However, patients also want to have a chat with them and come up with a decision together… " (P11)*.

#### Using mobile medical technologies

The utilization of mobile health technology as a medium for education has proven to be an attractive option for both patients and their families. Mobile health technologies optimize resource utilization, overcome spatial limitations, and enhance hospital communication to improve the efficiency of health education.

*“We are not native here, we’re getting discharged tomorrow. If something goes wrong at home, we cannot just rush to the hospital. In the information age, it would be ideal if we could communicate with doctors through mobile platforms promptly and conveniently.” (P6)*.

Patients anticipate that the hospital will provide long-term follow‐up care, establish an electronic record system for glaucoma patients, and take responsibility for follow-up management. Our hospital has implemented a comprehensive case management platform that encompasses patients’ personal information, surgical procedures, medication regimens, follow-up schedules and examination results as well as the Short Messaging Service. Two case managers can establish close connections with patients through the platform. One individual noted that the platform provides considerable convenience for ongoing follow-up efforts and serves to some extent as a motivator for continued engagement.

“*Case managers work closely with patients to help us understand the disease better… The patients were obligated to adhere to regular follow-up appointments upon enrolment in the system, which also served as a motivating factor for patient compliance.” (P1)*.

*“Moreover, health professionals were expected to provide patients with multiple convenient methods through Official Accounts or WeChat for self-management at home.” (P10)*.

### Theme 2: social support

#### Strengthen humanistic care

Patients anticipate that health professionals will engage in communication with them during outpatient visits or hospital stays, such as by soliciting questions or addressing any discomfort they may be experiencing. However, patients have reported that at times doctors prioritize ordering diagnostic tests or prescribing medication over addressing the subjective experiences of their patients.

*“Oh, by the way, it kind of bugged me when I went to the doctor and he was too busy staring at my Inspection Report instead of breaking them down for me.” (P10)*.

It would be highly appreciated if professionals proactively inquired about the patient’s well-being and assisted in addressing any challenges encountered during hospitalization. This leads to an increase in patient satisfaction.

*“Your hospital is excellent. The doctors attentively considered our input during daily rounds, Director Zhang provided invaluable assistance during my operation, and the nurses were attentive to my needs. It is just so tough being here without you, I cannot even imagine.” (P13)*.

#### Family support

Self-management often occurs within families, with spouses or children serving as primary caregivers. In regard to family support during illness, patients smile and praise the helpfulness of their spouses or children. Family members encourage them to engage in self-care activities such as medication adherence, proper eye care habits, and consistent follow-up.

*“My family members would criticize me for excessive use of my mobile phone and emphasize the importance of protecting my eyes.” (P4)*.

Seven patients reported that their spouses, children, or friends provided them with unwavering support as they faced the challenges of their illness. This emotional backing helped alleviate the pain caused by their condition and empowered them to confront these obstacles with a positive outlook.

*“As I expressed my fear of going blind as I grew older, my spouse reassured me that there was no need to be afraid and promised to always be by my side…I could not stay positive without his help, so I’m truly grateful for him.” (P3)*.

#### Peer support

Patients reported seeking out patient groups or communicating with other patients, through which they received information on glaucoma-related hospital resources and self-management skills that renewed their hope for treatment and reshaped their perception of the disease.

*“The fact that I have been to a lot of hospitals, but none of it has worked…I was referred here by a patient on WeChat groups.” (P10)*.

*“Today, I talked to a patient who was so naive that he believed his poor eyesight could be cured…So I told him that this disease was irreversible.” (P11)*.

Not only positive information but also negative information is transmitted through individuals who are seriously ill. The ability to make a certain psychological adjustment is required of patients; otherwise, they are susceptible to influence.

*“There are good and bad sides to having a group of patients because some people in the group can be negative and bring everyone down. I will try to take it easy and not let it get to me too much.” (P7)*.

#### Financial support

When questioned about their decision to forego surgery during the early stages of their illness, five patients with a melancholic expression conveyed that economic constraints compelled them to do so. Those responsible for child-rearing, eldercare, or academics often choose conservative treatment initially but may require surgery due to unstable IOP and progressive development of visual field loss. The financial burden is compounded by the heightened complexity of the procedure. Financial assistance, including reimbursement and charitable grants, is anticipated.

*“Initially, I refrained from undergoing surgery as I had the responsibility of supporting my two children. However, it was not until the impact on my productivity at the factory that I realized surgery was necessary… Do you know if the hospital offers any reimbursement options or accepts Medicaid?” (P5)*.

### Theme 3: psychological support

#### Managing negative emotions

Each patient reported experiencing adverse emotions such as fear, anger, anxiety, and depression during their illness due to elevated IOP, visual impairment, pain, and other symptoms.

*“…I have a tendency towards irritability, which is exacerbated by the onset of headaches and blurred vision.” (P2)*.

When informed that changes in mood could impact fluctuations in IOP, patients expressed some difficulty. Not all patients opt for interpersonal communication as a means of regulating their negative emotions. They have reported that such behaviour may exacerbate the distress of others, rendering it challenging for their loved ones to provide them with adequate support and alleviate their suffering. They prefer to shoulder the psychological weight of their illness on their own.

*“I am aware of the detrimental effects of anger and have made efforts to regulate my emotional responses.” (P9)*.

*“Due to their limited cultural background, it is understandable that my parents encountered difficulties in providing me with assistance…My classmates are all fresh out of college and busy with their own work, so unfortunately they cannot be of much help to me.” (P7)*.

#### Need to maintain self-esteem

The eye is a vital sensory organ of the human body responsible for visual perception and interpretation. Self-esteem is negatively impacted when the patient, due to conjunctival congestion and other ocular conditions, receives unfriendly looks from strangers while walking down the street.

*“I have been experiencing conjunctival congestion for a prolonged period. While I was being observed by a little boy on the subway, his mother intervened and instructed him to discontinue eye contact with me.” (P13)*.

Patients may also internalize the negative emotions of self-deprecation and self-discrimination, perceiving bias in others which can lead to a gradual reduction in social interaction.

*“My eyesight has gone downhill a lot since last year and my eyes are often irritated*, so *I lost interest when my friends invited me to travel with them.” (P4)*.

### Theme 4: daily living support

#### Guiding symptom monitoring

Eye care is a crucial aspect of postoperative recovery that requires careful attention. Professional guidance is requested by patients to avoid being helpless. With proper training, patients can learn to recognize bodily signals and handle any issues that arise with ease.

*“What is the mechanism behind postoperative inflammation of the eye? How should I manage elevated IOP? We need professionals to tell us in advance.” (P12)*.

#### Provides portable ways to monitor IOP

Pathological elevation of IOP is a risk factor for glaucoma, which requires patients to monitor IOP regularly. In most cases, regular monitoring of IOP in a hospital setting is necessary and poses greater challenges compared to the monitoring of blood pressure and blood sugar levels. Patients have expressed that inaccessibility due to the lack of IOP monitoring services in some hospitals or due to work-related reasons poses considerable challenges for IOP detection.

*“The iCare device, unlike a blood pressure monitor, is beyond my financial means. When I self-measured my IOP, I felt an increase in pressure. However, when measured at the hospital, it was within the normal range due to a time difference. It would be advantageous to be able to have real-time self-monitoring of IOP.” (P7)*.

## Discussion

The objective of this qualitative study was to gain insight into the challenges faced by patients in self-management and identify the type of support required for optimal self-management. There have been studies that have explored the disease experience of patients with glaucoma [26, 27], but the exploration of the fit between the patient’s needs and the support provided was particularly unique in this study.

Our findings show that all patients have expressed a desire for personalized information and care, with half of POAG patients expressing an interest in advanced surgical approaches, while the vast majority of PACG patients being more concerned about how they should care for themselves after surgery. Simply providing routine and standardized nursing education is insufficient for achieving long-term behavioural change, making it challenging to enhance self-management skills [28]. Thus, it is deemed crucial for healthcare professionals to provide education to individuals with chronic illnesses. Paula Newman-Casey’s Team [29, 30] has established the “Support, Educate Empower” personalized glaucoma coaching program pilot study, which is tailored to individuals based on their identification, laboratory results, and diagnostic testing. The program garnered high satisfaction rates among most participants and demonstrated substantial improvements in clinical outcomes and medication adherence. Moreover, patients expressed their reluctance to passively comply with healthcare professionals’ instructions and, instead, desired a collaborative partnership with physicians to discuss and jointly decide on treatment plans. The medical decisions that align most closely with patients’ preferences and needs are those made by informed patients who have been presented with disease information and the pros and cons of multiple treatment plans. This approach can enhance patient satisfaction and mitigate medical disputes to a certain extent. Therefore, assessing educational and health literacy beforehand is necessary for effective education. To effectively address patient priorities, tailored information support should be developed based on unique wishes, circumstances and conditions [31]. Professionals who provide sufficient health information support can help patients not only cope with the challenges of their disease but also reduce psychological anxiety and uncertainty. Moreover, mastering scientific and professional information is conducive to patients’ active participation in treatment decision making and cooperation with various treatment measures, thereby delaying the progression of the disease.

In the interviews, participants expressed the need for hospitals to provide uninterrupted and continuous services using digital resources. Electronic health(eHealth) is considered to be a technology that can replace nurse-caregivers because it is readily available and responsive. But it is doubtful whether eHealth will turn self-management into an expensive and unreported project. As a result, Calvin Kalun Or [32] has validated the safety and efficacy of eHealth in Patients with coexisting type 2 diabetes and hypertension. The study provided empirical support for the hypothesis that eHealth both improves patient self-care and meets patient self-safety. According to the previous data analysis of our hospital [33], the utilization of mobile platforms can substantially enhance the management of IOP in glaucoma patients and improve patient compliance with follow-up procedures. Our platform does not currently provide online consultation or virtual meeting capabilities. We expect to further advance the platform by using chat GPT to enhance multidisciplinary collaboration, enable multimodal human-machine interaction, intelligent Q&A and improve patients convenience during follow-up visits. In addition, some of the platform’s users are elderly or visually impaired, and we need to verify usability and acceptability [34, 35], considering whether the platform meets expectations and is easy to use for the users. Usability evaluation observes defects in the operation of the application, looks for major usability issues, fixes faults, iterates to find critical issues and continuously improves the application until performance targets are met.

Maintaining a good relationship with patients is key for health professionals to implement SMS [36]. The medical staff who provided care during their hospital stay were highly praised by the participants in the interview. However, due to the unclear definition of SMS among healthcare professionals, medical optimization is often prioritized over establishing patient relationships through effective communication [12, 17, 37]. A literature review [38] has indicated that the communication skills of nurses have a major impact on patients’ competence to self-manage their health behaviours. It is crucial to train professionals in supporting individuals with chronic illnesses in self-management. They can master a set of competencies in communication and psychological counselling through training at educational and interpersonal levels [39]. By gradually establishing a positive rapport through the use of open-ended inquiries, patients are made to feel understood and valued, resulting in a more humanistic approach to their medical care [40].

Glaucoma exerts a substantial psychological burden on patients and is strongly correlated with both anxiety and depression [2]. We found that those participants who were alone in hospital expressed strong concerns and anxieties during the interviews. Frequently, these patients expressed gratitude for our presence, as it alleviates the monotony during hospital stay. Our previous cross-sectional study [41] indicated that anxiety may have a considerable impact on the self-management of glaucoma patients and accelerate disease progression. Therefore, it is imperative that we give heightened attention to patients who are hospitalized without companionship. To alleviate anxiety, meditation, relaxing music, and autogenic training are viable options. Nurses can also help patients through motivational interviews and psychological counselling [42].

Previous research has indicated inadequate social support among glaucoma patients, leading to diminished well-being and quality of life [43]. To augment social support, chronic disease self-management programs have gradually incorporated peer and family member support interventions that have been effectively implemented across a range of chronic diseases [44–47]. In a scoping review of peer support in diabetes, the authors recommend that managers consider cultural competence as a cornerstone of implementing peer support. Care must be taken to recruit, train and retain peer supporters to adopt sustainable practices [44]. Only by acquiring self-care skills, cognitive behavioural skills, communication skills and other relevant courses can peer support workers and family members effectively integrate theory into practice and provide more professional support [48]. Analysis of the SMS program on HIV and breast cancer reveals that high-quality social and familial support systems can assist patients in mitigating the negative emotional effects of their illness while also guiding them towards a more proactive approach to treatment that improves their self-efficacy [47, 49, 50]. In addition to medical challenges, some glaucoma patients also face financial burdens. Research studies [51, 52] on cancer have found that economic toxicity can have a potential impact on patients’ medical adherence, mental health and quality of life. To help reduce the financial burden of cancer patients, health professionals train patients in skills and discuss how to manage symptoms effectively using less expensive preparations and call on the government to improve medical policies [51, 53]. Thus, we can be equipped with information regarding financial assistance programs or crowdfunding platforms to gain further insight into acquiring financial support. Government should also address the issue of employment for visually impaired people returning to work. This will alleviate the financial burden on patients and incentivize their active participation in treatment.

Symptom management is a very important part of self-management [54] involving prevention, assessment, nursing and targeted health guidance in relationship to symptoms. Glaucoma patients may have a variety of symptoms, one of the key aspects is IOP monitoring. However, both Goldmann applanation tonometry and noncontact airpuff tonometry necessitate hospital-based measurement of patients. ICare is a portable device, but its results can be easily influenced by central corneal thickness and require a more skilled measurement technique. Currently, many scholars are working on 24-hour IOP monitoring devices such as the implantable Biosensor [55], contact lens [56, 57], etc. We hope that soon, patients will have access to comfortable, inexpensive, and accurate portable devices. Additionally, appropriate patient-reported outcome measures (PROMs) [6, 58] can be utilized to attentively listen to patients, observe their symptoms, and accurately and dynamically evaluate their condition. Healthcare providers should focus on addressing the primary concerns of patients during different stages of treatment and offer targeted nursing guidance based on scientific evidence [58, 59].

This study has some limitations. The findings may not be generalizable to all patients with primary glaucoma in different settings, given that the study was conducted at a single centre in Wenzhou. Furthermore, patients’ support needs are continuously evolving, necessitating longitudinal interviews to dynamically comprehend their changing needs throughout the treatment process.

## Conclusion

This qualitative study involved interviewing with 20 primary glaucoma patients to investigate the self-management challenges and support needs of individuals living with this condition. The participants of this study expressed various challenges in terms of medical, emotional, and social tasks after being diagnosed with glaucoma: becoming an expert in glaucoma, managing negative emotions, adapting to daily life changes, and resuming social activities. In providing professional support, healthcare professionals should prioritize patients’ personalized health information needs and enhance their psychological and social support. Only by providing multidimensional and multifaceted self-management support can patients effectively cope with disease challenges and implement self-management.

### Electronic supplementary material

Below is the link to the electronic supplementary material.

Supplementary Material 1

## Data Availability

The datasets used and/or analysed during the current study are available from the corresponding author on reasonable request.

## Abbreviations

SMS: Self-management support
IOP: Intraocular pressure
PACG: Primary angle-closure glaucoma
POCG: Primary open-angle glaucoma
eHealth: Electronic health
PROMs: Patient-reported outcome measures
